# Construction and Regulation of Polymer@Silica Microspheres with Double-Shell Hollow Structures

**DOI:** 10.3390/molecules30040954

**Published:** 2025-02-18

**Authors:** Mingxiu Jiang, Yuanyuan Yang, Jiawei Feng, Zhaopan Wang, Wei Deng

**Affiliations:** 1 School of Material Science and Chemical Engineering, Harbin University of Science and Technology, Harbin 150040, China; 15603651853@163.com (M.J.); 13608827715@163.com (Y.Y.); 15776252556@163.com (J.F.); wangzhaopan2021@163.com (Z.W.); 2 Key Laboratory of Engineering Dielectric and Its Application, Ministry of Education, Harbin University of Science and Technology, Harbin 150080, China

**Keywords:** hollow structure, double-shell structure, composite microspheres, morphology control, compression resistance

## Abstract

Microspheres with well-defined hollow structures have been attracting interest due to their unique morphology and fascinating properties. Herein, a strategy for morphology and size control of hollow polymer@silica microspheres is proposed. Multilayer core–shell polymer microspheres, containing substantial carboxyl groups inside, evolve into microspheres with a 304 nm hollow structure after alkali treatment, which are used to construct hollow polymer@silica microspheres by coating the inorganic layer using the layer-by-layer (LBL) and sol–gel methods, respectively. The inorganic shell thickness of hollow polymer@silica microspheres can be adjusted from 15 nm to 33 nm by the self-assembled layers in the LBL method and from 15 nm to 63 nm by the dosage of precursor in the sol–gel method. Compared to the LBL method, the hollow polymer@silica microspheres prepared via the sol–gel method have a uniform and dense inorganic shell, thus ensuring the complete spherical morphology of the microspheres after calcination, even if the inorganic shell thickness is only 15 nm. Moreover, the hollow polymer@silica microspheres prepared via the sol–gel method exhibit improved compression resistance and good opacity, remaining intact at 16,000 psi and providing the corresponding coating with transmittance lower than 35.1%. This work highlights the morphology regulation of microspheres prepared by different methods and provides useful insights for the design of composites microspheres with controllable structures.

## 1. Introduction

Microspheres with well-defined structures and morphologies have attracted intensive attention and show prospects in many fields [1,2,3]. Hollow polymer microspheres have been widely used in biomedicine, catalysis, fillers, and waste removal, owing to their high specific area, light weight, and loading capacity [4,5]. However, the intrinsic low mechanical strength of polymers and their poor morphological stability block their further development. The design of core–shell structures not only allows the shell to protect and stabilize the core but also combines the merits of different components, thus exhibiting versatility, reactivity, and diverse physicochemical properties [6,7,8,9]. Therefore, the incorporation of hollow and hybrid core–shell structures into the construction of double-shell hollow structures may endow highly useful properties to microspheres, allowing them to meet the increasing demands of functionality [10,11].

Various methods have been developed to synthesize hollow polymer microspheres, such as the solvent extraction method [12,13], the template method [14,15], the self-assembly method [16,17], in situ polymerization [18,19], and the osmotic swelling method [20,21]. Nevertheless, their time-consuming nature and use of organic solvents, as well as the difficulty of morphology control and scalability, severely limit the application of many methods. Currently, the osmotic swelling method is the most industrially promising preparation technique, as it has the advantages of being environmentally friendly, easy to operate, and can control the hollow size. The typical process used in the osmotic swelling method involves the synthesis of microspheres with a designed core–shell structure and then the subsequent volume expansion of microspheres caused by ionization. The size distribution and morphology of hollow polymer microspheres play crucial roles in their properties and functionalities. For instance, Yang et al. regulated the particle size and volume expansion rate before and after alkali treatment by adjusting the dosage of emulsifier, treatment temperature, and pH value, and the resultant hollow polymer microspheres with a particle size of 489 nm and a volume expansion rate of 57% presented the best light-blocking performance for waterborne coatings [22]. Zhang et al. prepared hollow polymer microspheres with sizes in the range of 600 nm to 1000 nm using different types of emulsifiers, which were added into collagen fibers to construct a foamed animal skin material, exhibiting improved mechanical strength, porosity, thermal stability, and sound insulation performance [23].

Despite the notable progress made in the morphology control of hollow polymer microspheres, the low mechanical strength is still a problem to be solved. In addition to the characteristics of its counterparts, hollow silica particles are nontoxic, thermally stable, and exhibit high mechanical strength [24,25,26]. Layer-by-layer (LBL) assembly and the sol–gel process are commonly used methods for preparing hollow silica microspheres based on template-assisted strategies. The former is known as a versatile method for creating layered structures driven by electrostatic force, while the latter forms homogeneous and dense layers through hydrolysis and condensation. Whatever the method, the removal of the template is often accompanied by deformation, collapse, and even rupture of the silica microspheres [27].

Compared with single-shell hollow microspheres, hollow microspheres with two shells have more designability and diverse surface properties, and some techniques have been reported to fabricate such sophisticated structures [28,29,30]. Li et al. produced poly(methacrylic acid) (PMAA) hollow microspheres with asymmetric double shells by etching the silica core, and applying different cross-linking degrees to the PMAA double shells provided a hierarchical pH response, which can be used for controlled drug release; these structures displayed an overall improvement over conventional single-shell microspheres [31]. Lee et al. investigated hollow ZnSe@C microspheres with yolk–shell and double-shell structures comparatively, and the double-shell microspheres exhibited excellent electrochemical performances due to the synergetic effects of the structural and positional properties of the ZnSe nanocrystals embedded in the carbon shell [32]. However, most of the studies on double-shell microspheres have focused on inorganic@inorganic and polymer@polymer shell structures, while the construction of polymer–inorganic double shells is limited. Moreover, regulating polymer–inorganic double-shell microspheres with tailored morphologies remains a great challenge. By integrating the advantages of the polymer and inorganic shell layers, it is expected that the stability and properties of hollow microspheres will be significantly enhanced.

Herein, we present a successful attempt to fabricate polymer@silica microspheres with double-shell hollow structures. To achieve the desired structure, the evolution of the composition and morphology of the polymer microspheres prepared during the osmotic swelling process was investigated. Hollow microspheres with double shells were then obtained by directly armoring an inorganic layer onto hollow polymer microspheres via the LBL and sol–gel methods, respectively. Compared with the LBL method, the hollow polymer@silica microspheres prepared via the sol–gel method have a uniform and dense inorganic layer, enabling improved morphology retention and compression resistance. In addition, the hollow polymer@silica microspheres kept good opacity due to the refractive index difference between air in the hollow and the surrounding shells.

## 2. Results and Discussion

### 2.1. Morphological and Compositional Evolution of Multilayer Core–Shell Polymer Microspheres

**Figure 1:**
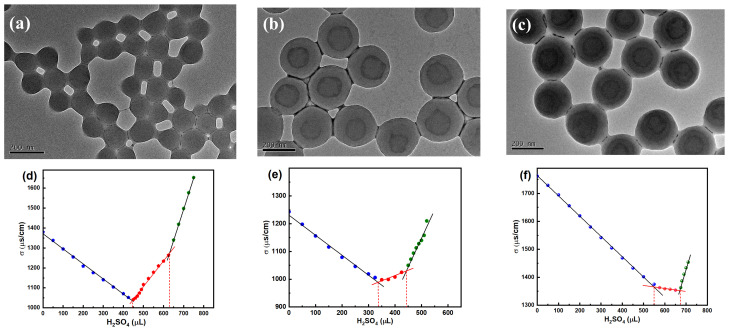
TEM images of microspheres synthesized at different stages: (**a**) core; (**b**) core/interlayer; and (**c**) core/interlayer/shell and their conductometric titration curves (**d**–**f**).

The morphologies and conductometric titration curves of polymer microspheres synthesized at different stages are shown in Figure 1. Owing to the employment of a soft monomer, BA, and a functional acid monomer, MAA, there is some adhesion between core microspheres (Figure 1a), which disappears after coating the interlayer and shell layer, and two- or three-layer structures can be clearly identified via TEM observation, as shown in Figure 1b,c. The sulfuric acid consumed during the intermediate period between the sharp decrease and sharp increase in conductivity seen in the titration curves (Figure 1d–f) represents the amount of -COOH bound on the surfaces of the polymer microspheres and freely existing in the aqueous phase (SFa) [22,33]; thus, the percentage of -COOH embedded inside the microspheres (PEa) can be deduced from the difference in the MAA dosage and SFa. Table 1 lists the size and PEa data of the core, core/interlayer, and core/interlayer/shell microspheres. As the seeded emulsion polymerization proceeds, the dried diameter (D^TEM^) and hydrodynamic diameter (D^DLS^) of the microspheres increase, and the hydration of carboxyl groups causes the D^DLS^ to be larger than its corresponding D^TEM^. The value of PEa also shows a trend of enhancement, indicating that the hydrophilic core containing carboxyl groups can be coated by the polarity transition intermediate layer and hydrophobic shell successively, which provides the possibility of forming hollow structures in the following alkali treatment process [34]. In addition, the polydispersity index stays relatively low throughout, indicating the size uniformity of the resultant microspheres.

### 2.2. Regulation of Hollow Structure Inside Polymer Microspheres

**Figure 2:**
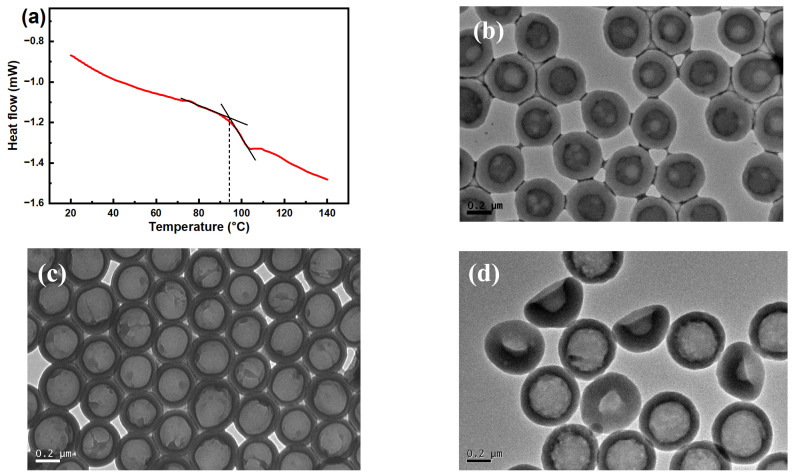
(**a**) DSC thermogram of multilayer core–shell polymer microspheres, and TEM images of alkali-treated microspheres with different pH values: (**b**) 8.5; (**c**) 9.3; and (**d**) 10.

Figure 2a shows that the glass transition temperature of the obtained multilayer core–shell microspheres is 93.8 °C, so the alkali post-treatment is performed at 95 °C, allowing base molecules to penetrates the shell to neutralize the carboxyl groups inside [35]. During the alkali treatment process, -COOH groups are neutralized to form -COONa groups; then, the hydration and osmotic pressure drive water molecules into the interior of the microspheres. Meanwhile, core chains with enhanced hydrophilic properties are more likely to move towards the aqueous phase. Also, the polymer chain segments of the shell layer have the mobile ability to generate large deformation at this temperature. Thereupon, the volume of the microspheres expands and void structures appear inside the swelling microspheres. The morphologies of the alkali-treated microspheres strongly depend on the ionization of carboxyl groups [22,36], and the effects of post-treatment at different pH values on the sizes and morphologies of the alkali-treated microspheres are listed in Table 2. When the pH value was adjusted to 8.5, the -COONa groups generated by neutralization were limited, and the hydration and osmotic pressure was not enough to drive the microspheres to an effective expansion, so the volume expansion rate was 188% and many small voids with average size of 72 nm appeared inside the microspheres (Figure 2b). Along with the increasing ionization degree (pH 9.3), the small voids inside the microspheres increased and tended to spontaneously aggregate to reduce the interfacial area with the polymer and lower the interfacial free energy, thus forming uniform microspheres with a higher volume expansion rate of 317% and one hollow structure with a size of 304 nm, as shown in Figure 2c. However, many ionized polymer chains may migrate out of the microspheres under excessive alkali conditions (pH 10), leading to collapse after drying, as the shell struggles to maintain the integrity of the spheres (Figure 2d).

### 2.3. Comparision of Preparation Techniques for Hollow polymer@silica Microspheres

**Figure 3:**
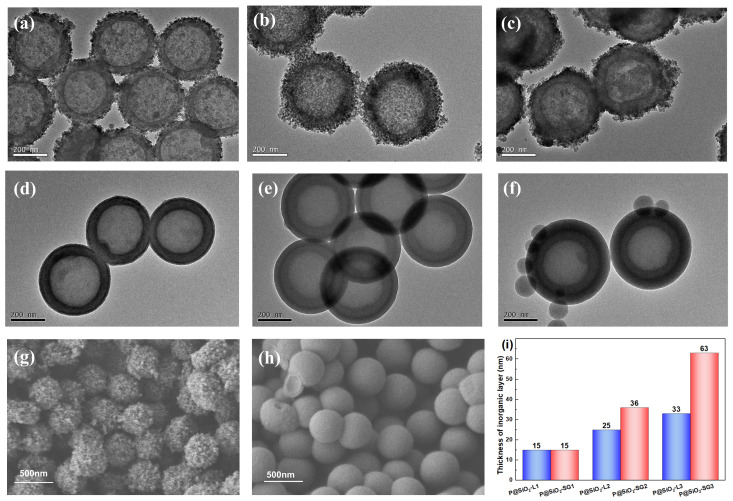
TEM images of hollow polymer@silica microspheres prepared via the LBL method and sol–gel method: (**a**) P@SiO_2_-L1; (**b**) P@SiO_2_-L2; (**c**) P@SiO_2_-L3; (**d**) P@SiO_2_-SG1; (**e**) P@SiO_2_-SG2; and (**f**) P@SiO_2_-SG3. SEM images of (**g**) P@SiO_2_-L2 and (**h**) P@SiO_2_-SG2, and (**i**) inorganic layer thickness of hollow polymer@silica microspheres.

The alkali-treated hollow polymer microspheres shown in Figure 2b are negatively charged with a zeta potential of −45.1 mV. After PEI modification, the zeta potential of the microspheres changes to +38.6 mV, which is suitable for the following absorption of negatively charged particles. The morphologies and sizes of microspheres prepared via the LBL and sol–gel methods are presented in Figure 3.

For the LBL method, the microspheres are alternatively assembled by polyelectrolyte and silicasol for different layers. The shell thickness of the microspheres increases from 15 nm to 33 nm as they absorb silicasol from one to three layers, and the morphology of the microspheres likewise changes in tandem. Specifically, P@SiO_2_-L1 has a relatively rough surface, since the microspheres only absorb one layer of silicasol, which is insufficient to thoroughly cover the microspheres (Figure 3a). The morphologies of P@SiO_2_-L2 become uniform after coating two layers of silicasol, but the structure remains rather loose, as shown in Figure 3b. As the coating of silicasol increases to three layers, obvious aggregation of silica nanoparticles appears on the surface of P@SiO_2_-L3 in the field of view (Figure 3c). Unlike the LBL method, the inorganic shell prepared via the sol–gel method can completely cover the hollow polymer microspheres, even if only in a very thin layer (Figure 3d), and the thickness of the inorganic shell layer is sensitive to the dosage of precursor [37]. When the TEOS used in the preparation of P@SiO_2_-SG2 is 2.5 times that of P@SiO_2_-SG1, the shell thickness of the resultant microspheres increases proportionally from 15 nm in P@SiO_2_-SG1 to 36 nm in P@SiO_2_-SG2 (Figure 3e); then, the shell thickness raises to 63 nm for P@SiO_2_-SG3. However, superfluous TEOS may result in a large number of unexpected free silicone gel particles in the system, as shown in Figure 3f. Moreover, in contrast to the loose and rough SiO_2_ layer prepared via the LBL method (Figure 3g), the inorganic shell obtained via the sol–gel method is compact and smooth (Figure 3h).

**Figure 4:**
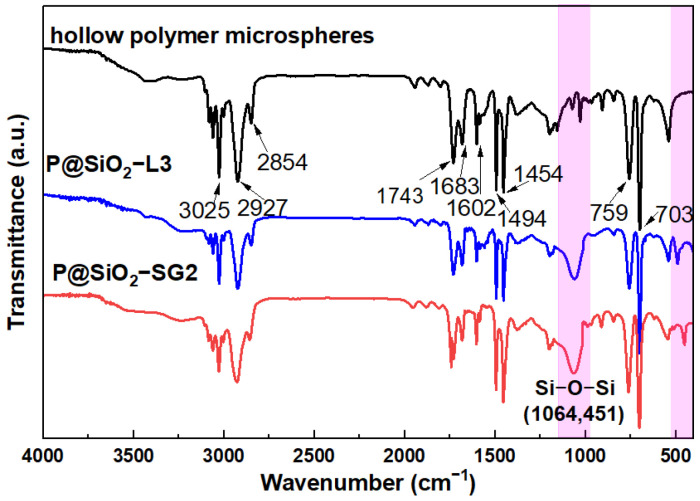
FTIR spectra of hollow polymer microspheres, P@SiO_2_-L3 and P@SiO_2_-SG2.

Figure 4 shows the FTIR spectra of hollow polymer microspheres and hollow polymer@silica microspheres prepared via the LBL and sol–gel method. There are obvious absorption peaks of the benzene ring, including a -CH stretching vibration at 3025 cm^−1^, overtones and combination bands at 1970 cm^−1^~1780 cm^−1^, skeletal vibrations at 1602 cm^−1^, 1494 cm^−1^, and 1454 cm^−1^, a -CH bending vibration at 759 cm^−1^, and a C=C bending vibration at 703 cm^−1^ [38]. The peaks at 2927 cm^−1^ and 2854 cm^−1^, 1743 cm^−1^, and 1683 cm^−1^ are associated with the stretching vibrations of -CH, C=O, and C=C from other copolymers. Furthermore, the coating with inorganic layers can be revealed by the newly originated peaks at 1064 cm^−1^ and 451 cm^−1^ in the spectra of hollow polymer@silica microspheres, which correspond to the stretching vibration and bending vibration of Si-O-Si, respectively [39].

**Figure 5:**
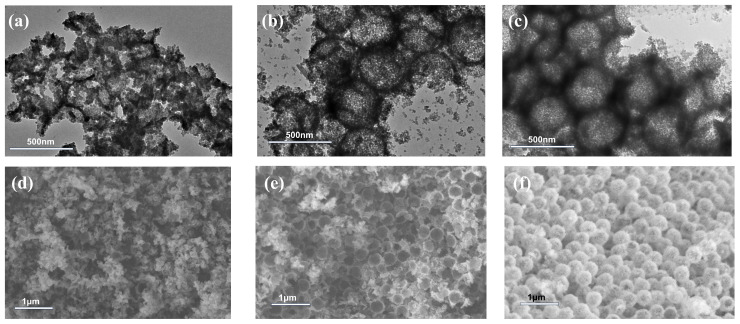
TEM images of hollow SiO_2_ obtained by calcining microspheres synthesized via the LBL method: (**a**) H-SiO_2_-L1; (**b**) H-SiO_2_-L2; (**c**) H-SiO_2_-L3; and corresponding SEM images (**d**–**f**).

**Figure 6:**
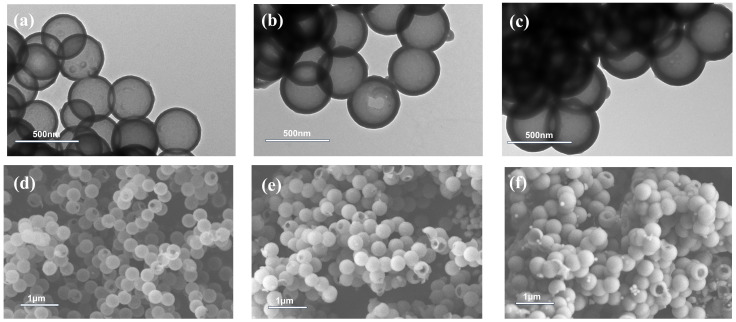
TEM images of hollow SiO_2_ obtained by calcining microspheres synthesized via the sol–gel method: (**a**) H-SiO_2_-SG1; (**b**) H-SiO_2_-SG2; (**c**) H-SiO_2_-SG3; and corresponding SEM images (**d**–**f**).

For further comparison of the inorganic shell formed by the LBL and sol–gel methods, the prepared hollow polymer@silica microspheres are calcined to remove the polymer layer, and the morphologies of the obtained hollow SiO_2_ are shown in Figure 5 and Figure 6.

By virtue of the weak van der Waals forces between the silicasol particles in the polymer@silica composite microspheres prepared via the LBL method, the removal of organic composition during calcination results in SiO_2_ particles falling off of the surface of the microspheres, making it difficult for the SiO_2_ layer to maintain the original spherical structure. When only one layer of silicasol particles is adsorbed, the calcined H-SiO_2_-L1 microspheres are completely broken (Figure 5a,d). When two layers of silicasol particles are adsorbed, most of the H-SiO_2_-L2 microspheres still collapse after calcination, and intact hollow microspheres can hardly be observed (Figure 5b,e). As the alternating adsorption of polyelectrolytes and silicasol grows to three layers, the strength of the shell layer is improved due to the increase in its thickness, and the hollow morphology of the calcined H-SiO_2_-L3 microspheres is preserved, but the shell structure is still very loose (Figure 5c,f).

Compared with the polymer@silica composite microspheres prepared via the LBL method, the inorganic layer formed via the sol–gel method has a uniform and dense structure, ensuring the complete spherical morphology of the microspheres after calcination. Even if the shell is as thin as 15 nm, and the calcined H-SiO_2_-SG1 microspheres are semi-transparent, almost no breakage or collapse can be observed (Figure 6a,d). With the increase in precursor dosage, the thickness of the SiO_2_ layer increases significantly (Figure 6b,d), indicating the enhancement of structure stability [40]. Unlike the uniform-size microspheres shown in Figure 6d,e, there are some small SiO_2_ particles deposited on the surface of H-SiO_2_-SG3 in Figure 6f, which is consistent with the TEM results of P@SiO_2_-SG3 shown in Figure 6c.

### 2.4. Compression Resistance and Opacity of Hollow polymer@silica Microspheres

**Figure 7:**
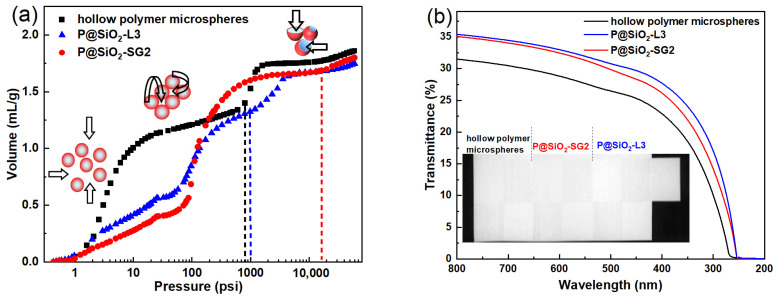
(**a**) Mercury intrusion curves of hollow polymer microspheres, P@SiO_2_-L3 and P@SiO_2_-SG2, and (**b**) transmittance of their corresponding coating. Insets (**a**,**b**) show the sample status and dry films on the Leneta cards, respectively.

The compression resistance is measured by the mercury intrusion method, during which mercury is injected into the sample gradually as the pressure increases. Three typical stages of the mercury intrusion curves are found for hollow microspheres, as represented in Figure 7a. The initial sharp rise stage under low pressure corresponds to the compression of incompact solid power, and the following relatively flat stage reflects the intrusion of mercury into the interspaces between each microsphere [41]. When the pressure exceeds the compressive strength of the microspheres, the hollow structure will be broken to allow the intrusion of mercury into the formed cavities, leading to a rapid increase in the cumulate volume of mercury with the increasing pressure. The mercury intrusion curves demonstrate that the compression strengths of the hollow polymer microspheres and P@SiO_2_-L3 are about 800 and 1000 psi, while the P@SiO_2_-SG2 stayed intact until the pressure was higher than 16,000 psi. The LBL method relies on electrostatic interactions between molecules and polymers to construct a coating layer by alternately depositing silica gel and PEI with opposite charges. The sol–gel method is used to form a chemically uniform coating layer by hydrolysis and condensation of the precursor, as well as the subsequent gelation process. Compared with the covalent interactions in the sol–gel method, the non-covalent interactions in the LBL method have dynamic reversibility, and defects are easily generated under applied force, which may act as a stress raiser and can be responsible for the relatively low compressive strength of the P@SiO_2_-L3 microspheres. The dense and integrated inorganic layer formed by the sol–gel method enables the intrinsic mechanical properties of SiO_2_ to be better utilized, thus enhancing the strength of the hollow polymer@silica P@SiO_2_-SG2 microspheres. In addition, the injection volume of mercury into hollow polymer@silica microspheres is less than that into hollow polymer microspheres, which can be ascribed to the reduction in the proportion of the hollow volume after coating inorganic layers.

Figure 7b presents the opacity of different coatings with the synthetic hollow polymer microspheres, P@SiO_2_-L3 and P@SiO_2_-SG2, as pigments, respectively. Due to the difference in refractive index between air in the hollow structure and the surrounding polymer or polymer@silica shell, light is effectively scattered, contributing to opacity. In the wavelength range of 200~800 nm, the coating containing hollow polymer microspheres displays transmittance below 31.5%, and the maximum transmittance increases to 35.1% and 35.4% after coating the inorganic layer via the LBL and sol–gel methods, respectively. This is because the number of hollow polymer@silica microspheres is much lower than that of hollow polymer microspheres at the same dosage in the coating due to the increased particle size and density. Despite this, the hollow polymer@silica microspheres cater to the optimum particle size (400~600 nm) and hollow size (250~300 nm) determined according to light scattering theory [42], thus exhibiting good opacity.

## 3. Materials and Methods

### 3.1. Materials

Methyl methacrylate (MMA), butyl acrylate (BA), methacrylic acid (MAA), styrene (St), divinylbenzene (DVB, 55% mixture of isomers), sodium dodecyl sulfate (SDS), ammonium persulfate (APS), sodium hydroxide (NaOH), ammonia (25–28%), ethanol (EtOH), and tetraethoxysilane (TEOS) were purchased from Shanghai Macklin Biochemical Technology Co., Ltd. (Shanghai, China). All the monomers were distilled under reduced pressure and APS was purified by recrystallization twice in water before use. Ethylene glycol dimethacrylate (EGDMA, 98%) and polyethyleneimine (PEI, 99%, M_w_ = 10,000) were provided by Shanghai Aladdin Biochemical Technology Co., Ltd. (Shanghai, China). Silicasol (30 wt%, 10 nm, pH = 9) was supplied by Guangzhou Suixin Chemical Co., Ltd. (Guangzhou, China).

### 3.2. Synthesis of Hollow Polymer Microspheres

The hollow structures were formed inside polymer microspheres by performing alkali post-treatment on the multilayer core–shell polymer microspheres, which were synthesized by multistep seeded emulsion polymerization, and the typical recipes for preparing core–shell polymer microspheres are shown in Table 3.

First, 5 wt% core monomers, mixed with H_2_O (90 g), APS (0.10 g), and SDS (0.025 g), were polymerized at 80 °C for 40 min. Next, the residual core monomer mixture and APS aqueous solution (10 g, 3 wt%) were simultaneously added dropwise into the reactor at 80 °C within 4.5 h; then, the reaction system was heated to 90 °C and maintained for 30 min to obtain core latex. After diluting the core latex (10 g) with H_2_O (25 g), the interlayer monomer mixture and APS aqueous solution (5 g, 1 wt%) were simultaneously added dropwise into the diluted core latex at 80 °C within 1 h to coat the interlayer onto the core microspheres. For the shell preparation, St, BA and DVB, as well as APS aqueous solution (10 g, 1 wt%), were simultaneously added dropwise into the resultant core/interlayer latex at 90 °C within 2 h and continuously reacted for 30 min. Lastly, the pH of the core–shell emulsion was adjusted with 5 wt% NaOH aqueous solution and alkali post-treatment was performed at 95 °C for 3 h, followed by drying to form hollow polymer microspheres.

### 3.3. Synthesis of Hollow polymer@silica Microspheres

**Figure 8:**
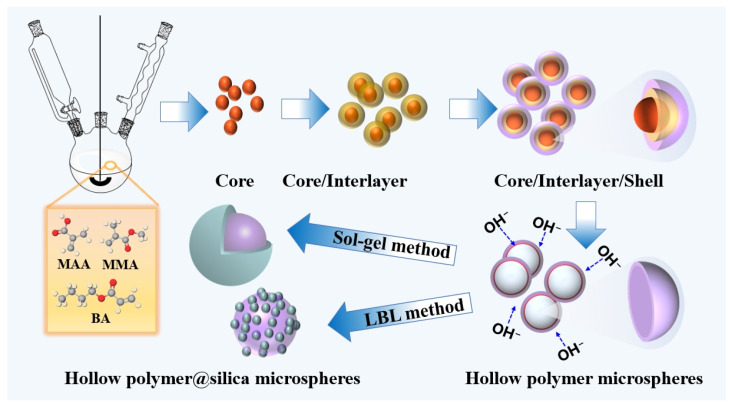
Schematic illustration of the preparation process of hollow polymer@silica microspheres.

The whole preparation process of the hollow polymer@silica microspheres is illustrated in Figure 8. Initially, PEI aqueous solution (100 g, 0.1 wt%) was used to modify the polymer hollow microsphere emulsion (33 g, 1.5 wt%). After continuous agitation for 6 h, the excessive PEI was removed by three cycles of centrifugating/washing with H_2_O at 9000 r/min. Then, silica was coated on the surface of the modified polymer hollow microspheres using the sol–gel and LBL methods, respectively. The resultant hollow polymer@silica microspheres were air-calcined at 500 °C for 4 h in the muffle furnace to obtain hollow SiO_2_ microspheres.

For the LBL method, PEI-modified polymer hollow microspheres (0.33 g) were redispersed in NaCl aqueous solution (80 g, 5.85 wt%), and then silicasol (2.5 g) was slowly dropped into the dispersion. Herewith, PEI and silicasol were successively adsorbed onto the polymer microspheres via electrostatic interaction. After each layer was wrapped, the microspheres were collected by three cycles of centrifugating/washing with H_2_O at 9000 r/min. The resultant hollow polymer@silica microspheres, prepared with different layers of silicasol (1, 2, and 3), were recorded as P@SiO_2_-L1, P@SiO_2_-L2, and P@SiO_2_-L3, respectively. The corresponding hollow SiO_2_ microspheres obtained after calcination were referred to as H-SiO_2_-L1, H-SiO_2_-L2, and H-SiO_2_-L3, respectively.

For the sol–gel method, PEI-modified polymer hollow microspheres (1 g), H_2_O (16 g), and EtOH (64 g) were added into a flask and sonicated for 20 min. After using ammonia to adjust the pH of the system to 9, TEOS was dropped into the flask within 1.5 h and the reaction was maintained for 24 h. Finally, hollow polymer@silica microspheres were obtained through three centrifugation/resuspension cycles and were dried in an oven at 80 °C for 24 h. The resultant hollow polymer@silica microspheres prepared with different amount of TEOS (2.5 g, 6.2 g and 11.2 g) were recorded as P@SiO_2_-SG1, P@SiO_2_-SG2, and P@SiO_2_-SG3, respectively. The corresponding hollow SiO_2_ microspheres obtained after calcination were referred to as H-SiO_2_-SG1, H-SiO_2_-SG2, and H-SiO_2_-SG3, respectively.

### 3.4. Characterization

Transmission electron microscopy (TEM, JEOL, JEM-2010, Tokyo, Japan) and scanning electron microscopy (SEM, JEOL, JEM-7401F, Tokyo, Japan) was used to observe the morphology and dried diameter (D^TEM^) of the microspheres. The hydrodynamic diameter (D^DLS^), polydispersity index, and zeta potential (ζ) of the microspheres was measured on a Zetasizer 3000HS (Malvern, Malvern City, UK) at a scattering angle of 90°. The percentage of -COOH embedded inside the microspheres (PEa) was determined by conductometric titration using conductometer (Leici, DDS-307, Shanghai, China) as follows. The original latex was diluted to a solid content of 0.3% and the pH value was adjusted to 11.5 ± 0.02 using NaOH aqueous solution (1.5 wt%); then, the variation in latex conductance (σ) was recorded during titration with H_2_SO_4_ aqueous solution (1.0 wt%). The glass transition temperature (T_g_) of the core–shell polymer microspheres was analyzed by a differential scanning calorimeter (DSC, Shimadzu, DSC-60, Kyoto, Japan) with a heating rate of 10 °C/min. The chemical structures of samples were characterized by a Fourier transform infrared spectrometer (FTIR, ThermoFisher, NicoletIS10, Waltham, MA, USA) in the wavenumber range of 500~4000 cm^−1^. A mercury intrusion porosimeter (Micromeritics, Autopore IV 9510, Norcross, GA, USA) was used to test the compression resistance of the hollow microspheres. The transmittance of the coating, which was obtained by mixing the hollow microspheres with acrylate emulsion at a mass ratio of 1:4, was tested using a UV–visible spectrometer (Shimadzu, UV-2450, Kyoto, Japan) in the wavelength range of 200~800 nm.

## 4. Conclusions

In this work, polymer@silica microspheres with double-shell hollow structures were prepared by combining the osmotic swelling method with the LBL or sol–gel methods, which involved using seeded emulsion polymerization to fabricate multilayer core–shell microspheres, alkali treatment to form hollow insides, and the subsequent coating of the inorganic layer. As the seeded emulsion polymerization proceeded, the size of the microspheres and the percentage of carboxyl groups embedded inside increased, and then the morphologies of the microspheres evolved from core–shell structures to become porous, hollow, and then collapsed successively following alkali treatment. Controlling the self-assembly of layers in the LBL method and the dosage of precursor in the sol–gel method allowed us to tune the thickness of the silica layer in the hollow polymer@silica microspheres. The silica layer formed via the LBL method had a loose and rough structure, which contributed little to the compression resistance of the microspheres and made it difficult to preserve the hollow structure after calcination. In contrast, the silica layer formed via the sol–gel method had a uniform and dense structure; thus, the corresponding hollow polymer@silica microspheres remained intact both under 16,000 psi pressure and after calcination. Moreover, all the hollow microspheres displayed good opacity, and the transmittance of the corresponding coating was lower than 35.4%.

## Figures and Tables

**Table 1:** Size and percentage of -COOH embedded inside of microspheres synthesized at different stages.

Microspheres	D^TEM^ (nm)	D^DLS^ (nm)	Polydispersity Index	PEa (%)
core	126	199	0.0237	67.71
core/interlayer	178	294	0.0933	73.11
core/interlayer/shell	251	429	0.0758	90.26

**Table 2:** Effects of post-treatment at different pH values on the size and morphology of microspheres.

pH Value of Post-Treatment	Morphology	D^TEM^ (nm)	Average Void Size (nm)	Volume Expansion Rate (%)
8.5	porous	357	72	188
9.3	hollow	404	304	317
10.0	collapse	468	-- *	-- *

* Due to the collapse of microspheres, the void size and diameter of microspheres can not be measured accurately, and the volume expansion rate can not be obtained.

**Table 3:** Typical recipes for multilayer core–shell polymer microspheres.

Ingredients	Core (g)	Interlayer (g)	Shell (g)
MMA	16.50	3.66	0
MAA	10.04	0.42	0
BA	14.70	0	0.45
EGDMA	0.25	0	0
St	0	1.02	11.84
DVB	0	0	0.45
SDS	0.03	0	0
APS	0.40	0.05	0.10
H_2_O	100	30	10
Core latex	0	10	0

## Data Availability

Data are contained within the article.
